# Evidence for sodium-rich alkaline water in the Tagish Lake parent body and implications for amino acid synthesis and racemization

**DOI:** 10.1073/pnas.2003276117

**Published:** 2020-05-11

**Authors:** Lee F. White, Kimberly T. Tait, Brian Langelier, Elizabeth A. Lymer, Ana Černok, Tanya V. Kizovski, Chi Ma, Oliver Tschauner, Richard I. Nicklin

**Affiliations:** ^a^Department of Natural History, Royal Ontario Museum, Toronto, ON M5S 2C6, Canada;; ^b^Department of Earth Sciences, University of Toronto, Toronto, ON M5S 3B1, Canada;; ^c^Canadian Centre for Electron Microscopy, McMaster University, Hamilton, ON L8S 4M1, Canada;; ^d^Lassonde School of Engineering, York University, Toronto, ON M3J 1P3, Canada;; ^e^Division of Geological and Planetary Sciences, California Institute of Technology, Pasadena, CA 91125;; ^f^Department of Geoscience, University of Nevada, Las Vegas, NV 89154

**Keywords:** Tagish Lake, framboidal magnetite, atom probe tomography, amino acid

## Abstract

Understanding the timing and mechanisms of amino acid synthesis and racemization on asteroidal parent bodies is key to demonstrating how amino acids evolved to be mostly left-handed in living organisms on Earth. It has been postulated that racemization can occur rapidly dependent on several factors, including the pH of the aqueous solution. Here, we conduct nanoscale geochemical analysis of a framboidal magnetite grain within the Tagish Lake carbonaceous chondrite to demonstrate that the interlocking crystal arrangement formed within a sodium-rich, alkaline fluid environment. Notably, we report on the discovery of Na-enriched subgrain boundaries and nanometer-scale Ca and Mg layers surrounding individual framboids. These interstitial coatings would yield a surface charge state of zero in more-alkaline fluids and prevent assimilation of the individual framboids into a single grain. This basic solution would support rapid synthesis and racemization rates on the order of years, suggesting that the low abundances of amino acids in Tagish Lake cannot be ascribed to fluid chemistry.

The Tagish Lake meteorite is a unique piece of the asteroid belt, a highly brecciated ungrouped carbonaceous (C2) chondrite with minimal terrestrial alteration following retrieval of the main mass within days (1–3). Due to the pristine nature and recovery of the Tagish Lake meteorite, its insoluble and soluble organic constituents such as amino acids, amines, and hydrocarbons have been thoroughly studied to better understand the evolution of prebiotic life in our solar system (e.g., ref. 4). Of particular interest is the rate of racemization, or the natural process of amino acids changing chirality from one hand (L) to the other (D), on the parent body. Within Tagish Lake, large l-enantiomer excesses (L_ee_ < 59%) of aspartic and glutamic amino acids are juxtaposed by a nearly racemic (D ≈ L) alanine population (4). This variation has been ascribed to amplification of an initial l-enantiomer excess during aqueous alteration (4). It has been postulated that racemization can occur quite quickly depending on several factors, including the temperature and pH of the aqueous solution (4, 5). While alteration products are numerous within the asteroidal meteorite record, particularly in CM-type chondrites (6), direct isotopic and mineralogical evidence of the early liquids responsible for this alteration is largely absent (7).

Micrometer-scale three-dimensional assemblages of interlocking 110- to 680-nm-wide magnetite crystals have been observed in both clasts and matrix of the Tagish Lake meteorite (2, 8). While the magnetic properties of these features suggest formation within isolated droplets of water on the parent body (8), some grains appear to pseudomorph after pyrrhotite, resulting in a hexagonal shape to the agglomerated crystals (2). In both scenarios, intensive fluid interaction is required to form the observed structures—an observation supported by identical magnetite features in the CI meteorites Orgueil, Alais, and Ivuna (9), for which an aqueous origin has also been ascribed. It has previously been proposed that the 0- to 3-nm-thick amorphous boundary layers of these nanocrystalline assemblages contain remnant residue of their parent solution (8). However, the chemistry of these nanometer-scale domains is nearly impossible to resolve with micrometer-scale analytical techniques. In this study, we use atom probe tomography (APT) to isolate and measure the chemistry of these amorphous intergrain domains to yield insights into the acidity and composition of the oldest water in the early solar system and better constrain the rate of amino acid synthesis and racemization on the Tagish Lake parent body.

A framboidal magnetite (Fe_3_O_4_) cluster, measuring ∼50 μm in total diameter (Fig. 1*A*), was located within a thin section of the Tagish Lake meteorite (accession number M52292 in the Royal Ontario Museum [ROM] collection). Initial characterization and imaging work was conducted using a scanning electron microscope (SEM) at the California Institute of Technology and a Raman spectrometer at the ROM. The larger feature comprises multiple <10-μm-diameter clusters separated by regions of amorphous carbonaceous material. While these features are largely spherical, some domains appear subhedral as a result of deformation during collision with a neighboring spherule, suggesting heterogenous timing of formation for individual spherules. Each cluster is, in turn, defined by a network of hundreds of rounded, interlocking <680-nm magnetite spheres which appear tightly packed in rounded clusters, and loosely packed (with elevated abundances of interstitial carbonaceous material) in deformed and subspherical features (Fig. 1*B*). Six APT microtip specimens were prepared using a Zeiss NVision 40 focused ion beam SEM (FIB-SEM) system at the Canadian Centre for Electron Microscopy, McMaster University. Five tips were prepared at ambient conditions, while the final stages of polishing for a single microtip were conducted under cryogenic conditions to minimize possible volatile loss. APT samples were analyzed using a CAMECA 4000X HR, operating in laser-pulsed mode. Of the six tips, four failed during analysis, likely as a result of inconsistent evaporation between carbonaceous and magnetite regions. However, datasets R47_02212 (microtip prepared under ambient conditions) and R47_02314 (cryogenically prepared microtip) yielded datasets in excess of 10 million total measured ions (10).

**Fig. 1.**
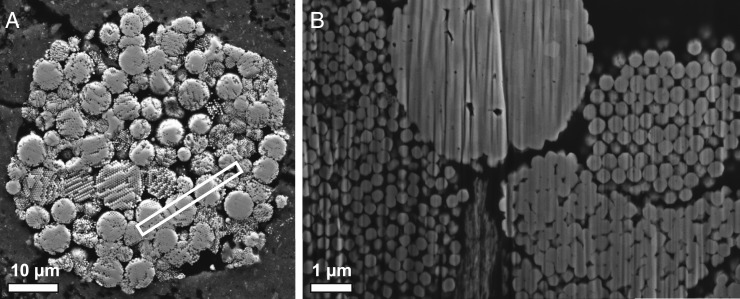
Secondary electron images of magnetite framboids in Tagish Lake. The larger spherical to hexagonal structure within the thin section (*A*) is constructed of numerous framboidal aggregates, as imaged during FIB analysis of the sample (*B*). The abundance of carbonaceous material within the framboids is correlated with the tightness of packing, with deformed regions incorporating higher quantities of carbon. For reference, the lift-out location for atom probe microtip preparation is highlighted in *A*.

Dataset R47_02212, which captures a boundary between interstitial carbonaceous material and a magnetite framboid, reveals an ∼25- to 45-nm-wide Mg- and Ca-enriched boundary between the domains. The boundary does not appear to contain any further nanofeatures. Within the carbon-rich region, semiquantitative analysis also shows H and Si, and minor amounts of Na and Mn, to all be present in relatively higher concentrations than the adjacent magnetite grain, correlating with a drop in Fe and O abundances. In comparison, dataset R47_02314 captures a curved boundary between two magnetite framboids of similar orientation (confirmed by the alignment of an apparent [011] pole in the APT ion density map). Data for the magnetite domains are more reliably quantifiable. Their composition is almost pure Fe and O (∼99.5 atomic % total) and are found to be chemically homogenous throughout. The ∼30-nm-wide subgrain boundary contains elevated abundances of homogenously distributed Mg and Mn, along with Na segregated into clusters of ∼10-nm diameter (Fig. 2). Na clusters contain ∼30 atomic % Na, significantly more enriched than the surrounding magnetite grains (0.014 wt % Na). Considering this segregation of Na as being composed of Na^+^ cations, the observation of a comparable drop in Fe, representing Fe^+^ ions, acts to balance the localized charge within these clusters. Additionally, incompatible elements (principally Mg, Mn, and Na) also define a dislocation loop in direct association with the boundary (Fig. 2), suggestive of a high density of dislocations and point defects within the material.

**Fig. 2.**
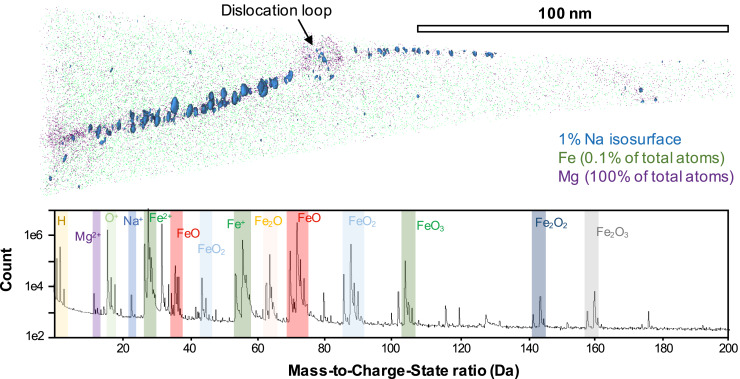
APT analysis of a decorated subgrain boundary between magnetite framboids in the Tagish Lake meteorite (cryogenically prepared microtip R47_02314). A curved, Mg-enriched boundary, which runs between two Fe_3_O_4_ (magnetite) grains, is densely decorated by ∼10-nm-wide Na-rich clusters. Dislocation loops, decorated by Mg, Na, and Mn, can also be observed in association with the boundaries. For reference, the mass-to-charge spectrum for the microtip is shown (0 Da to 200 Da), with major peak families highlighted, including the Na, Mg, and Fe peaks integrated into the reconstruction.

The acidity of the fluids responsible for alteration on the Tagish Lake parent body has been constrained to pH 7 to 10 based on computer simulations assuming a starting CM material (11), although this is hard to reconcile with the observed magnetite framboid structures, which typically require a more acidic solution (pH 5.4 to 6.8) to prevent buildup of surface charge and subsequent amalgamation of grains into a single magnetite mass (8). However, the presence of Ca and Mg cations as interstitial coatings on the framboids, as observed in microtip R47_02212, would facilitate a surface charge state of zero within the more basic fluids previously predicted (11), preventing coagulation into a single grain and producing the uniform, well-ordered colloidal structures observed here. As a result, these APT analyses act to constrain the pH of the formative fluid on the Tagish Lake parent body to be more alkaline in nature. Furthermore, the abundance of clustered Na on subgrain boundaries trapped within the magnetite framboids strongly supports an excess of sodium in the parental fluid, which would have been segregated to the boundaries during growth of the magnetite framboids and clustered during deformation of the material, as highlighted by the dislocation loop in contact with the clustered surface.

When modeling racemization timelines, a neutral pH is often assumed in calculations. However, with the discovery of Ca- and Mg-enriched boundary layers and segregated Na clusters between magnetite framboids formed in aqueous solution, we show that this solution would be of a higher pH than originally suspected (8). This more basic solution would provide interconversion rates that are much quicker than that of a neutral pH (6), supporting rapid racemization of amino acids on the Tagish Lake parent body. For example, aspartic acid in warm (80 °C) alkaline (pH 9) conditions would racemize within ∼176 d [assuming published rate constants (4) and D/L ratios (6)], significantly faster than at neutral conditions (4). Additionally, alkaline solutions also support faster synthesis of amino acids (11), although this observation fails to reconcile with the low abundance of amino acids in the Tagish Lake meteorite (<5,400 parts per billion) (12, 13). Thus, we show that the abundances of amino acids are not limited by fluid chemistry, and, instead, Tagish Lake must be deficient due to the absence of another key component (such as aldehydes or ammonia) for amino acid synthesis and racemization. This component is clearly abundant within other meteorites such as Murchison, which boasts an abundance of l-enantiomers and similar amino acids (14). Future sample return missions should thus prioritize Murchison-like parent bodies to ensure a high content of organic matter, samples of which would be prime candidates for nanoscale chemical analysis using APT.

## Data Availability

Raw data files (.pos and .rrng) to support this study can be accessed through the associated Open Science Framework project (DOI 10.17605/OSF.IO/TJV8W).
